# Supplementary material for: Effectiveness of bimanual coordination tasks performance in improving coordination skills and cognitive functions in elderly
Source: PLoS One. 2020 Mar 4;15(3):e0228599. doi: 10.1371/journal.pone.0228599 (PMC7055901; doi:10.1371/journal.pone.0228599)
Supplement: Attachment — Submitted filename: Response to Reviewers.docx [file pone.0228599.s002.docx]

I am very grateful to the reviewers for their comments. I have considered each one and have done my best to respond accordingly. In the revised manuscript I have marked my content-related corrections in blue. Below I address each comment.

**Reviewer #1:**

*“More previous studies should be addressed in the introduction part. The gap between previous studies and this research should be clearly described.”*

In ‘Introduction’ section there has been added a text that addresses this issue: “Standardized trainings equipped with adequate measurement variables can both serve rehabilitation purposes and as an indicator of neurodegenerative diseases. Restoring or improving coordination skills after trauma or in the face of chronic consequences of stroke, can be a goal of performance of such training [19,20]. Many devices have been developed to train the upper limbs or to support the training of people after a stroke (for review see vanDelden et al. [20]). The devices differ considerably in terms of their mechanical and electromechanical complexity from relatively simple to complex ones. However, the overwhelming majority of these devices are not adapted to measure the performance. Evaluation of the effects of the rehabilitation training has been done only by means of assessment of the improvement of the post intervention functionality.

Few publications have presented training and their evaluation by quantitative measures. Training sessions were performed once [21,22] or repeated in two, three or five days [15,23-28]. Tasks included in trainings required operating sensors by forearm movements that forced cursor movements on the monitor. Sensors were in a form of handles that slid in parallel along the track from side to side in front of the body [23,27], rotated discs [21,25,26,28] or joysticks [22,24]. The monitor display showed Lissajous figure and tracing lines that had been produced by the participant movements [21,23,27], a blue target line and white dot which follow that line [25,26,28] or two white dots on a black background [24]. In Hoff et al. [15] study participants observed a learning sequence consisting of a 15-letters array on the monitor. Participants pressed the corresponding keys on a keyboard with a corresponding finger when a specific letter occurred. All those studies looked at changes in performance measures in subsequent training sessions. However, the training included only one task and the possible modifications concerned only the number of repetitions and speed of the performance. Given that involvement of brain regions depends on the task characteristics [29] and that task characteristics can affect performance progress in the training of bimanual coordination, there is a need of such a training that would be equipped with various tasks, differentiated according to characteristic features, e.g. trajectory on which the cursor moves, speed of movement, type of the cursor tracking. “ (Starts at page 5).

*“The study completed successive training during 2-3 days aims to the same group, it’s common sense to get some positive effects. However, What about the duration of effects? Is it necessary to conduct long-period observation?”*

In ‘Discussion’ section there has been added a text that addresses this issue: “Some of studies examined the effect of retention interval in one week [21,24,25,27], one month [27], eight weeks [21] and six months [28]. All those studies showed that performance measures kept their values after the retention interval regardless the type of coordination task that was under study. In present study there were six trainings sessions implemented, which is more than in other studies. Study of Hoff et al. [15] in which retention was calculated as the difference in the amount of measure between successive training sessions showed lack of differences in retention between young and old people. Therefore, based on the previous results it was assumed that the retention effect will be sustained and it was not tested. To conduct long-period observation would gain more knowledge on the subject. However, it seems it would not provide different results compared to previous studies.” (Starts at page 17).

*“The Participants‘s attributes are varied, which would affect the research conclusions.”*

In ‘Study limitation’ section there has been added a text that addresses this issue: “To the best of our knowledge, there is no research that would provide information on the effects of gender or obesity on learning bimanual coordination. However, even if gender is an important factor in the ability to bimanual coordination learning, it does not affect the present study, as the study group had the same number of women and men. It was demonstrated that limb domination affects bimanual coordination, since people with restricted domination show better results [50], and lateral specialisation has a negative effect on the performance of symmetric tasks [51]. Also the results might be biased by diseases, especially of neurodegenerative origin, which means that the study conclusions can refer only to healthy, older people.” (page 21).

*“The manuscript is hard to read, cause the fonts and layout are inappropriate.”*

It has been corrected

**Reviewer #2:**

*“The topic of this paper is important and the methods sound. The results are meaningful.”*

We thank Reviewer for this comment.

*“For “51 and 58%”, it will be better to be changed to “51-58%” or “51% and 58%”.”*

It has been corrected.

*“For “mean reaction time”, the definition of reaction time is needed, which the authors could refer to the following paper. And does the “mean” means the average reaction time of all participants? It is not clear.
[1] “Examining the safety of trucks under crosswind at bridge-tunnel section: A driving simulator study”, Tunnelling and Underground Space Technology, 2019, 92, 103034.”*

It has been clarified and the publication has been added. The text reads now: “For the measurement of cognitive functions, attention assessment tests Cognitrone (COG) and the test of information processing Reaction Test (RT) were selected. The following indicators were counted in the COG test: Time of correctly rejected (MTR) (s); Time of correctly accepted (MTA) (s). The following indicators were counted in the RT test: Reaction time [31], which is a measure of reaction speed in response to relevant stimuli (MRT) (ms); Motor time, i.e. measure of the speed of movement in planned action sequences (MMT) (ms). Each indicator was calculated as an average value of series of measurements for each study participant.” (page 12).

*“There should be some references for Wilcoxon test.”*

References to Wilcoxon test has been added: “The Wilcoxon Sign Test [32] was used for this purpose.” (page 13).

*“In the conclusion part, the author could list some major findings in this paper.”*

Conclusion section has been modified according to the Reviewer’s comment.
